# Variant ribosomal DNA is essential for female differentiation in zebrafish

**DOI:** 10.1098/rstb.2024.0107

**Published:** 2025-03-06

**Authors:** Tim V. Moser, Donna M. Bond, Timothy A. Hore

**Affiliations:** ^1^ University of Otago, Dunedin 9054, New Zealand

**Keywords:** sex determination, zebrafish, ribosomal DNA, ribosome heterogeneity, CRISPR

## Abstract

The ribosome consists of protein and RNA components. Deletion of genes encoding specific ribosomal proteins has revealed that heterogeneity in the ribosome must exist in vertebrates; however, this has not been tested for ribosomal RNA (rRNA). In zebrafish (*Danio rerio*), the ‘45S-M’ ribosomal RNA-encoding locus undergoes massive extrachromosomal amplification during oocyte growth and ovary differentiation and is distinct from the regular ribosomal DNA (rDNA) locus encoding somatic rRNA (45S-S). Although the 45S-M rDNA locus falls within the only described sex-linked region in multiple wild zebrafish strains, its role in sexual differentiation is unclear. We used CRISPR–Cas9 gene editing to alter 45S-M rDNA sequences in zygotes and found that although there was no effect on growth or male development, there was dramatic suppression of female differentiation. Males with edited 45S-M rDNA produced phenotypically normal sperm and were able to fertilize eggs from wild-type females, with resulting embryos once more displaying normal development. Our work supports the hypothesis that specialized 45S-M rDNA is the elusive apical sex-determining locus in zebrafish and that this region represents the most tractable genetic system to date for studying ribosomal RNA heterogeneity and function in a vertebrate.

This article is part of the discussion meeting issue ‘Ribosome diversity and its impact on protein synthesis, development and disease’.

## 1. Introduction

The ribosome is a macromolecular machine that translates genetic information from messenger RNA (mRNA) into protein [1]. Ribosomes are themselves a complex of ribosomal RNA (rRNA) molecules and many ribosomal proteins, with the rRNA at the catalytic centre and ribosomal proteins helping provide structure [2,3]. Since the experiments of Brenner *et al*. [4], ribosomes have largely been thought of as uniform and non-specialized [4–7]. Nevertheless, mutation and deletion of specific ribosome proteins are not necessarily lethal and can cause very specific phenotypic alterations—something incompatible with the hypothesis that all ribosomes are homogeneous and act in the same manner. An early example of this was from *Drosophila* with the *Minute* phenotype spectrum, where mutant flies are characterized by delayed development featuring short and narrow thoracic bristles, with mutations in specific ribosome proteins driving the phenotype [8,9]. More recently, it has been shown that ribosomes containing alternative ribosome proteins preferentially translate distinct sub-pools of mRNA in mouse embryonic stem cells [10] and that male germ-cell-specific ribosome proteins like RPL39L are essential for spermatogenesis and male fertility but have little effect on female and non-sex related phenotypes [11].

While ribosome protein heterogeneity is becoming better understood, much less is known about rRNA diversity. There are a number of reasons for this, including the fact that ribosomal DNA (rDNA) encoding rRNA is repetitive and poorly assembled even in model genomes [12]. For example, 45S rDNA encodes three of the four eukaryote rRNA types (18S, 5.8S and 28S rRNA) [13]) and are found spread over five distinct loci in humans and mice [14,15]. Allelic variants of 45S rDNA have been discovered, some of which have unique epigenetic and transcriptional characteristics; however, it has not been conclusively shown how these variants are distributed within discrete rDNA clusters [16–18]. Perhaps because of the inability to distinguish specific rDNA regions or the assumption that all ribosomal DNA is essential to life, genetic disruption of individual rDNA loci in vertebrates has never been achieved. Therefore, it has so far not been possible to be conclusive about the function of specific rDNA clusters.

**Figure 1.**
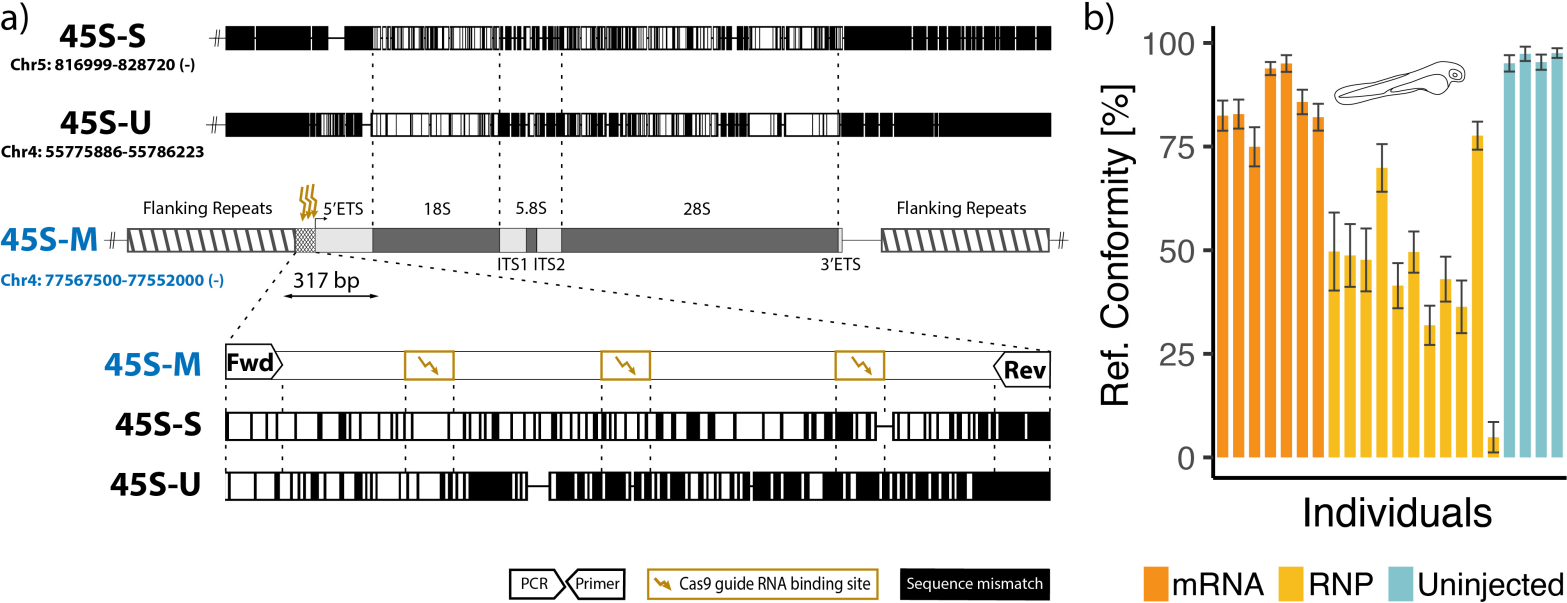
Guided Cas9 mutation of the zebrafish 45S-M rDNA variant. (*a*) Map of the targeted maternal 45S (45S-M) ribosomal DNA (rDNA) region and its homology to other 45S variants, based on the GRCz11 reference genome. The 45S-M unit is found on the tip of chromosome 4 (centre), while the 45S-U variant is found elsewhere on chromosome 4, with 45S-S located on chromosome 5 (upper). Shared nucleotide identity between the S and U variants and the M variant are displayed in white, with mismatches in black. In the lower panel, a 317 bp region adjacent to the 45S-M transcription start site is depicted. This region contained the binding sites of the three co-injected Cas9-associated single guide RNAs (sgRNAs), specifically chosen to harbour at least some degree of sequence mismatch compared with the other 45S variants (black). Flanking this region are PCR primers capable of amplifying all target sites simultaneously. (*b*) Degree of conformity between the reference and sequenced 45S-M promoter resulting from zygote injections of the three sgRNAs in conjunction with Cas9 as a ribonucleoprotein or an mRNA template. Black bars represent margin of error resulting from the number of paired reads.

Nevertheless, there are some variant rDNA loci that strongly imply functional specialization. In zebrafish, there are at least three distinct 45S rDNA types, organized in separate tandem repeats [19]. We and others have shown that only one of these, a ‘maternal’ 45S (45S-M, also known as fem-rDNA), is involved in striking DNA amplification in the female germline [19,20]. The onset of 45S-M rDNA amplification in the female germline coincides with the earliest recognizable oocyte stages, 1A and 1B, which are marked by an increase in cell volume, the onset of meiosis and the formation of a follicle surrounding the germ cell [21]. From stage 1B onwards, the number of nucleoli—the sites of rRNA synthesis—increases from around four to roughly 1500 by stage 4 [22]. Germ cells purified from around this developmental timepoint show a massive increase in 45S-M rDNA, such that up to one in five DNA fragments sequenced belong to this locus [20]. It is presumed that 45S-M amplification occurs via a rolling circle mechanism, mediated by recombination of the loci at unique long terminal repeats flanking the 45S-M central region [23]. rDNA amplification has been documented in other species, like *Xenopus*, where extrachromosomal copies have successfully been imaged using electron microscopy [24]. Interestingly, the zebrafish M and S variants of 45S show a high degree of sequence divergence, with a pairwise identity of only 86% at the rRNA elements (figure 1). Even highly conserved regions such as the peptidyl transferase centre, the GTPase-associated centre and the sarcin–ricin domains display a limited number of mismatches [19]. Together, this implies that 45S-M has a highly unusual developmental role in the female germline, albeit undescribed to date.

Here, we show that disruption of the variant rDNA locus 45S-M is possible using guided Cas9 endonuclease mutation in zygotes. Surprisingly, editing of 45S-M causes no apparent malformation, allowing normal, undelayed development of embryos into adulthood even in highly modified individuals. Yet, 45S-M-modified individuals develop almost exclusively into phenotypic males, capable of producing fertile sperm. By identifying a variant rDNA cluster that is fundamental to biological processes such as feminization of the gonad, we predict the zebrafish 45S-M is an ideal model system from which to explore the wider implications of rRNA heterogeneity.

## 2. Material and methods

### (a) Animal handling

Zebrafish (AB strain) were maintained under standard Otago Zebrafish Facility operating procedures, and genetic manipulation was performed under approval GMD101727. Microinjection into the cytoplasm of the first cell using pulled glass needles was performed on eggs obtained through natural pairwise breeding. For each set of injected eggs, a comparable quantity of uninjected eggs belonging to the same clutch was maintained as a reference. Individuals <7 days post-fertilization (dpf) were maintained at 28.5°C in E3 medium (5 mM NaCl, 0.17 mM KCl, 0.43 mM CaCl_2_ and 0.40 mM MgCl_2_). Older individuals were housed in 3.5 l plastic tanks with recirculating flow-through (Tecniplast, Italy), while maintaining pH 6.9–7.6, 300–600 mS conductivity and 28°C, with a 14/10 h dark/light cycle including twilight phases. Fish were fed dry food (ZMS, UK) or live *Artemia nauplii* thrice daily ad libitum. To obtain whole fish lysate, individuals ≤29 dpf were euthanized by submersion in ice water for >20 min [25]. Fin clips, sperm and images of individuals >29 dpf were obtained under fresh Tricaine solution-induced (150 mg l^−1^; NaOH adjusted to pH 7.0) short-term anaesthesia [26].

### (b) Production of guide RNAs for genetic manipulation

Annotations for the 45S-M region were based on Breit *et al*. [23], using the GRCz11 reference genome [27]. Guide RNAs targeting 45S-M immediately upstream of the 5' external transcribed spacer (5′ ETS) sequence (figure 1) were designed using the IDT CRISPR–Cas9 guide RNA design Web tool [28]. The 5′ nucleotide of the resulting 20 bp target CRISPR RNA (crRNA) sequences was replaced with a 5′-G (if not already selected by the design tool), such that the T7 transcription initiation site is a 5′-GG [29].

DNA templates for the complete 45S-M single guide RNAs (sgRNAs) included two partially complementary overlapping oligonucleotides (termed ‘overgos’), one being specific to the target of interest and the other ‘universal’ (graphical illustration in electronic supplementary material, figure S1; template sequences are listed in electronic supplementary material, table S1). The target overgo includes the T7 promoter sequence (5′-GCGTTAATACGACTCACTATAG-3′), the 20 bp target sequence of interest (with a 5′-G; see above) and the first 34 bp of the *trans*-activating CRISPR RNA (tracrRNA) scaffold sequence (5′-GTTTTAGAGCTAGAAATAGCAAGTTAAAATAAGG-3′). The universal overgo (in reverse order) includes a 30 bp sequence complementary to the tracrRNA scaffold sequence in the target overgo (underlined above; 5′-CCTTATTTTAACTTGCTATTTCTAGCTCTA-3′), the remaining 42 bp of the tracrRNA scaffold sequence (5′-GCACCGACTCGGTGCCACTTTTTCAAGTTGATAACGGACTAG-3′) and a 4 bp polyT sequence (to produce a polyA sequence for the sgRNA). For each 45S-M-sgRNA, the target overgo and universal overgo were mixed (0.125 µM of each oligo) with 4 µl of 10× NEBuffer 2 (New England Biolabs, B7002) and nuclease-free water to 38 µl and placed under the following annealing conditions: 95, 70, 60, 50, 40, 30 and 25℃ for 5 min each before ramping at a rate of 0.1℃ s^−1^ to the next lower temperature. With tubes still in the PCR machine, the following components were added to initiate extension/fill-in of the 5′ overhang annealed oligos: 0.8 µl 10 mM dNTPs (0.2 mM final) and 1.2 µl Klenow fragment 3′ → 5′exo- (New England Biolabs, M0212). Reactions were mixed with a P20 pipette and incubated at 37℃ for 30 min. The resulting sgRNAs were cleaned-up using a 2× ratio of solid-phase reversible immobilization (SPRI) carboxyl-coated Sera-Mag Magnetic SpeedBeads (Cytvia, 45152105050250) diluted in standard PEG buffer (18% w/v polyethylene glycol 8000 (PEG), 1 M NaCl, 10 mM Tris (pH 8.0), 1 mM EDTA, 0.05% v/v Tween-20) [30].

An additional sgRNA, targeting green fluorescent protein (GFP) [31] and used as a mock injection (target not present in the AB strain), was synthesized using the sgRNA cloning system designed by [32]. Briefly, a pair of oligonucleotides was annealed such that overhangs on the resulting DNA molecule are compatible with directional cloning into the pDR274 vector cut with *Bsa*I (New England Biolabs, R3733). The resulting pDR274:GFPsgRNA vector was digested with *Dra*I (New England Biolabs, R0129) and used as an *in vitro* transcription template.

All sgRNA templates were *in vitro* transcribed using the MAXIscript T7 Kit (Invitrogen, AM1314) as per the kit manufacturer’s instructions with one deviation. Incubation time for the transcription step was extended to 5 h as initial trials conducted with a shorter incubation time did not produce the expected yield. The resulting sgRNAs were stored at −80ºC.

Ribonucleoproteins (RNPs) were formed on the day of injection by combining 1 µl of 20 µM recombinant Cas9 protein (Alt-R™ S.p. Cas9 Nuclease V3; IDT, 1081058) in Opti-MEM buffer (Gibco, 31985062) with 5.6 µl of 118 ng µl^−1^ (approx. 3.6 µM) sgRNA at 21°C for 5 min. Each of the three 45S-M-targeting RNPs was formed separately before being combined in equivolume amounts. Per embryo, approximately 1.84 nl of vehicle containing roughly 60 pg each of the three sgRNAs and 902 pg Cas9 as RNP was injected.

Initial testing of editing capability was performed with Cas9 mRNA *in vitro* transcribed using the mMESSAGE mMACHINE^®^ T7 Ultra Kit (Invitrogen) from plasmid MLM3639 [32] digested with *Pme*I (New England Biolabs, R0560). For injections with 3× sgRNAs, 0.8 µl each of 57 ng µl^−1^ sgRNA were combined with 2 µl of 300 ng µl^−1^ Cas9 mRNA, resulting in approximately 250 pg Cas9 mRNA and 19 pg sgRNA (each) per 1.84 µl injection. For single-guide injections, a calculated 19 pg of sgRNA together with 83 pg Cas9 mRNA.

### (c) Phenotyping and sperm collection

For the purpose of phenotyping sex, anaesthetized 100 dpf fish were momentarily positioned in a six-well plate and photographed . Female sex was inferred from the presence of a cloacal papilla [33]. Sperm was collected from fish identified as males at 171 dpf. Sperm was released by gently prodding the gonadal region of prospective males with a smooth, blunt spatula. A small aliquot of each sperm sample was collected in immobilizer buffer (5 mM NaCl, 100 mM KCl, 100 mM CaCl_2_ and 100 mM HEPES), transferred to distilled water and briefly assessed for appearance and activity under a compound light microscope (ZEISS Primostar3).

### (d) DNA extraction

Samples were lysed in TNES buffer (100 mM Tris (pH 8.0), 25 mM NaCl, 10 mM EDTA and 10% w/v SDS) with 5 µl proteinase K (20 mg ml^−1^) for 24 h at 55°C, with light shaking. For sperm samples, TNES was supplemented with 5% v/v β-mercaptoethanol as a reducing agent [34]. DNA was extracted from the entire lysate or a fraction, depending on sample size, following a modified version of the Bio-On-Magnetic-Beads (BOMB) protocol [30]. In short, TNES lysate (20 µl) was mixed with 6 M guanidinium isothiocyanate buffer (20 µl), 40 µl of solid-phase reversible immobilization (SPRI) beads suspended in 1× TE buffer (10 mM Tris (pH 8.0) and 1 mM EDTA) and 80 µl of isopropanol. Beads were magnetically separated on a neodymium magnetic rack for approximately 5 min until the solution was clarified. The supernatant was removed, and the DNA bound to the magnetic beads was washed once with isopropanol and twice with 70% ethanol. Following air-drying of the beads, DNA was eluted with 10 mM TE.

### (e) Amplicon sequencing for genetic editing assessment

Detection of off-target effects was limited to monitoring of embryo viability. For on-target effect assessment, a 317 bp amplicon (as determined using the GRCz11 reference genome; [27]) encompassing the target sites of the three 45S-M-specific sgRNAs was amplified. The majority of amplicons were assessed using a modified ‘Lange-handle’ two-step, four-primer PCR approach [35] (electronic supplementary material, figure S2). During the first round of amplification, target-specific primers terminating in ‘Lange-handle’ overhangs at the 5′ end were added in the following PCR mix: 10−25 ng DNA, 1× High Fidelity buffer with 1.5 mM MgCl_2_, 0.2 mM of each dNTP, 0.5 µM of each primer and 0.02 U µl^−1^ of Phusion polymerase (New England Biolabs, M0530L). PCR parameters during this initial amplification were: 95°C for 2 min, 27 cycles of 98°C for 20 s, 62°C for 10 s and 72°C for 20 s; final extension occurred over 10 min at 72°C before a cooldown to 10°C. Half of the resulting PCR product was cleaned up and size-selected using 0.9× SPRI beads diluted in standard PEG buffer, washed with 70% ethanol and eluted in 10 µl nuclease-free water. Next, 2−5 µl of this eluate was indexed using P5 and P7 primers (indexed Truseq-type oligos for Illumina sequencing) containing an added linker sequence complementary to the aforementioned Lange handle. Reaction mix and cycle conditions during this second step were as above except for a reduction to five cycles, each ending with an extended time of 40 s at 72°C. Amplicons from initial trials were assessed following a modified ‘ligation of adaptors’ protocol described by [36] using the same target primer sequences without ‘handles’. All amplicons were pooled in equimolar amounts, gel-extracted using a MinElute Gel Extraction Kit (Qiagen) according to the manufacturer's instructions, and sequenced on an iSeq100 (Illumina) to generate 150  bp paired-end reads. Primer sequences can be found in electronic supplementary material, table S1.

To quantify the extent of editing achieved, a custom bioinformatics pipeline testing conformity between sequenced amplicons and the reference sequence was designed. Following phred >20 quality trimming via TrimGalore [37], on-target reads were tested for localized indels at the target sites of sgRNA#1 and #4 (± 5 bp of protospacer adjacent motif −3 bp). To detect large deletions spanning from one target site to the next, it was tested if the 3′ end of reads matched positions on the reference sequence more than 151 bp from where their start matched the reference (i.e. the PCR primer site). During this preliminary classification step, one or more indel variants were assigned to each read. The final category for each read pair was determined based on certain indel variants taking precedence over others (for further illustration see electronic supplementary material, figure S7).

Only samples covered by >150 read pairs (see electronic supplementary material, figures S8 and S9) with a calculated margin of error below 5% (see statistics and data visualization) were considered in the final analysis. Raw sequencing data and custom 45S-M indel variant identification scripts are available at [38] .

### (f) Detection of 45S-M amplification

To assess 45S-M rDNA levels as an indicator for femaleness, DNA extracts of whole fish lysates were processed using post-bisulfite adaptor tagging (PBAT) [39]. Bisulfite conversion of the resulting DNA was performed using the EZ Methylation Direct Mag Prep kit (Zymo, D5044), but reducing the reaction volume to one-quarter of that stated in the protocol (to allow for all reaction and clean-up steps to be performed in PCR tubes). The bisulfite-treated DNA was consequently processed following a modified PBAT [40,41] protocol and a library indexing procedure described in [39]. Resulting libraries were pooled in equivolume amounts and were 151 bp paired-end sequenced on the iSeq100 (Illumina). Raw sequencing data were quality-checked in FastQC v. 0.11.9 (https://www.bioinformatics.babraham.ac.uk/projects/fastqc/) and phred >20 quality-trimmed via TrimGalore [37]. PBAT-specific mapping of sequencing reads to the zebrafish reference genome (GRCz11) and CpG methylation calling were performed using Bismark v. 0.19.0 [42]. Sample-specific distribution of CpG calls across the genome was assessed with the mapped sequence data analyser SeqMonk (v. 1.48.0, Babraham Bioinformatics). For sex assessment purposes, the genome was divided into 64 067 windows of 21 kb length, and the proportion of calls mapping to the window surrounding 45S-M (Chr4: 77 553 001−77 574 000) was calculated. For samples to be included in further analysis, a conversion efficiency threshold of >97% and a minimum number of CG calls above 10 000 were chosen. Samples with more than 1% of reads identified as identical replicates through SeqMonk were also rejected. PBAT read and CG call numbers of all samples considered in the final analysis are shown in electronic supplementary material, figure S9.

### (g) Statistics and data visualization

Margins of error (*ε*) were calculated based on the number of specific paired reads (*n*) for a sample according to the standard asymptotic estimator *ε* = 𝑧√(𝑝(1−𝑝)/*n*), at a confidence interval of 99% (*z* = 2.326348). Differences in indel variant diversity between fin clips and sperm were quantified by calculating Shannon entropy values (*H*(*X*)), according to the formula *H*(*X*) = −Σ *P*(*X* = *x*)log_2_(*P*(*X* = *x*)). ‘*P*(*X* = *x*)’ is the probability that a read will be a specific indel variant out of all the indel variants present in the sample. Higher entropy values suggest increased diversity. A Wilcoxon rank-sum test was used to calculate the significance of differences between groups ( * *p* ≤ 0.05, ** *p* ≤ 0.01, *** *p* ≤ 0.001 and **** *p* ≤ 0.0001). Graphs were plotted using the ggplot2 (v. 3.0.0) plugin within R (https://www.r-project.org/). Assembly of some subfigures and icon overlays were done using Adobe Illustrator (Adobe, 2023). To visualize the homology between the three 45S variants, the Geneius (Dotmatics) mapper for alignment was used before redrawing the final graphic in Illustrator.

## 3. Results

To test the function of the 45S-M rDNA variant in zebrafish, we altered its sequence using sgRNA-guided Cas9 mutation. To minimize interference with 45S-S expression, active in somatic tissue, as well as a third rDNA variant (45S-U, with currently unknown expression profile), particular attention was paid to the degree of homology between the three variants (figure 1a). Of the 17 sgRNAs targeting 45S-M (electronic supplementary material, table S1) that we initially designed and tested, most were rejected during initial feasibility assessment owing to repeatedly producing low yield during *in vitro* transcription, failure to amplify only the targeted 45S variant when testing for mutation, or, in the case of sgRNAs targeting the 45S-M flanking repeat, producing products of highly variable length. Fortunately, three closely grouped sgRNAs designed to target the promoter region of 45S-M were found to cause detectable editing and could be assayed using a single PCR product preferentially amplifying 45S-M over other 45S variants—hence we focused experimentation on these (figure 1a).

The level of 45S-M modification was assessed by amplicon sequencing and quantification of reads matching a 45S-M consensus sequence. When supplying Cas9 mRNA together with all 3 sgRNAs, we found 85.28 ± 2.67% (mean ± s.e.m., *n* = 7) of reads matched the reference sequence in fish sacrificed 48 h after injection (figure 1b), compared with 96.35 ± 0.65*%* (*n* = 4) in an uninjected control. In contrast, when we injected the same cocktail, but instead used Cas9 RNP, we found 45.51 ± 5.74% (*n* = 11) of reads matched the reference. Considering the significant improvement in editing efficiency, all subsequent editing experiments were performed using Cas9 RNP, rather than Cas9 mRNA.

Throughout the initial trial phase to validate the sgRNAs and Cas9 as mRNA or RNP, injected zygotes displayed a reduction in survivability and an increase in aberrant phenotypes. However, this was consistent across all controls, including non-functional injections such as a single sgRNA without Cas9. The three guide RNP 45S-M treatment increased the rate of malformations at 2 dpf from 3.15% (*n* = 174) in uninjected, to 8.7% (*n* = 182) in the injected group without a clear trend towards a specific morphology (electronic supplementary material, figure S3). By 4 dpf, 83.9% of uninjected and 60.99% of injected individuals exhibited normal growth (electronic supplementary material, figure S3).

### (a) 45S-M editing prevents germline ribosomal DNA amplification but does not affect growth

While 45S-M editing appeared to have limited effect on early development, we wanted to test if it played a significant role during later development, especially following the generally rapid growth period ending approximately 30 dpf [43]. Importantly, we found no relationship between 45S-M rDNA editing and fish growth. During the first experiment, individuals with <75% reference conformity (i.e. edited fish) grew to a standard length of 8.37 ± 0.30 mm (mean ± s.e.m., *n* = 35), while fish showing >75% reference conformity (untreated or unaffected) grew to 8.23 ± 0.23 mm (*n* = 43) by 29 dpf (electronic supplementary material, figure S4). During the second experiment, edited 29 dpf individuals were 8.37 mm, and those with >75% reference conformity were 9.00 ± 0.19 mm (*n* = 40, including mock-treated specimens). Moreover, we did not observe any visible phenotypic differences between 45S-M edited and control fish, implying that 45S-M rDNA operates in a specialized manner, not outwardly visible at this developmental timepoint.

Prior experiments using whole-genome bisulfite sequencing on isolated germline cells showed 29 dpf fish have extensively amplified 45S-M rDNA [20]. To test if amplification of 45S-M could be detected on whole fish lysate using the same technique, we sampled wild-type AB fish at a range of developmental time-points up to 29 dpf (electronic supplementary material, figure S5). We found considerable 45S-M amplification (up to 1.3% of total reads) in presumptive 29 dpf females using whole fish lysate. By contrast, the median proportion of 45S-M at unamplified stages between 4 and 18 dpf was 0.214% (*n* = 115).

**Figure 2.**
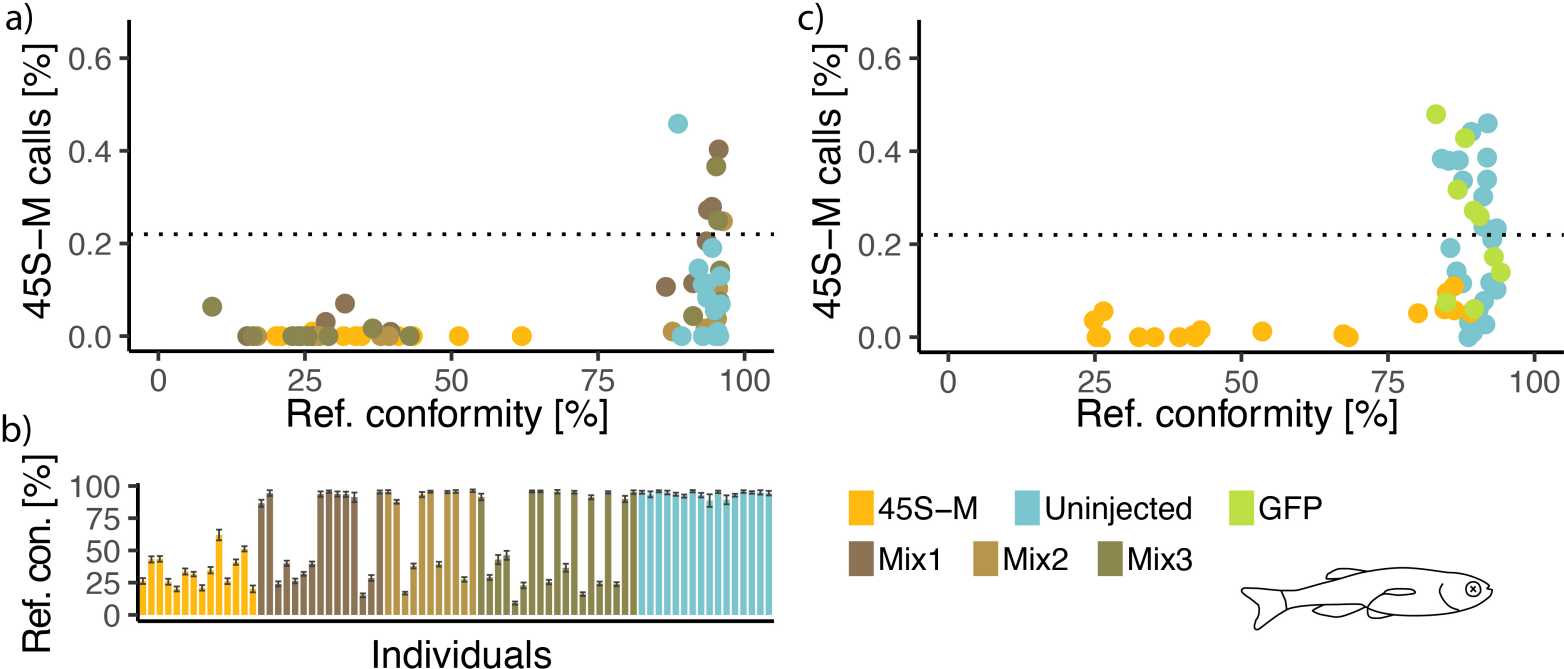
Female-associated 45S-M ribosomal DNA levels at 29 dpf. (*a*) Percentage of CG calls mapping to the window containing the 45S-M locus out of 64 067 × 21 kb windows across the genome in relation to the degree of conformity between the reference and sequenced 45S-M promoter. (*b*) The extent of reduction in 45S-M promoter reference conformity allows clear separation between injected and uninjected specimens even within tanks containing a mixture of both. Black bars represent margin of error resulting from the number of paired reads. (*c*) Results from an independent repeat of the experiment, introducing an additional mock control in the form of Cas9 targeting reen fluorescent protein (GFP) (not present in the AB strain fish). Entirety of figure based on whole fish lysate DNA. *(a,c*) The dotted line represents the unamplified 45S-M call frequency observed in lysate from 4 to 18 dpf fry.

We then asked if 45S-M edited fish have altered amplification at 29 dpf—none of those injected with 45S-M sgRNA and Cas9 RNP showed 45S-M read proportions beyond the non-amplified baseline (figure 2*a*, yellow), contrary to uninjected control fish (figure 2*a*, light blue). This finding was corroborated with measurement of 45S-M editing from the same tissue lysates—only fish with unmodified 45S-M sequence showed evidence of 45S-M amplification. Global DNA methylation levels did not differ between modified and unmodified samples (electronic supplementary material, figure S4c). It is known that tank-specific effects, such as rearing density or food availability, can significantly skew sex ratios in zebrafish, potentially confounding our results. To control for this possibility, three tanks were stocked with a mix of injected and uninjected fish (figure 2*b*, brown). Again, only those fish with unedited 45S-M showed rDNA amplification in 29 dpf whole fish lysate (figure 2*a*, brown).

An independent repeat of this experiment, conducted several weeks later using different breeders, further confirmed these results, albeit at slightly lowered editing efficiency (figure 2*c*). To ensure that the reduction in 45S-M amplification represented a specific effect rather than a side-effect of injection or sgRNA presence, we introduced an additional control group where GFP (not present in these fish) was targeted (figure 2*c*, green). This mock injection group experienced no reduction in 45S-M reference conformity, and five of nine individuals showed high levels of amplification, likely indicating a female phenotype.

### (b) Disruption of the 45S-M promoter causes phenotypic masculinization

In order to confirm 45S-M editing drives masculinization of zebrafish, we examined edited and control fish at 100 dpf, a timepoint where the presence of female genital papilla becomes an obvious phenotype separating males and females [33]. Among uninjected fish from three separate clutches, 64% (*n* = 46) exhibited female papilla. In contrast, within the injected 45S-M group (*n* = 45) from the same clutches, only a single fish was identified as possessing female papilla (figure 2a).

**Figure 3.**
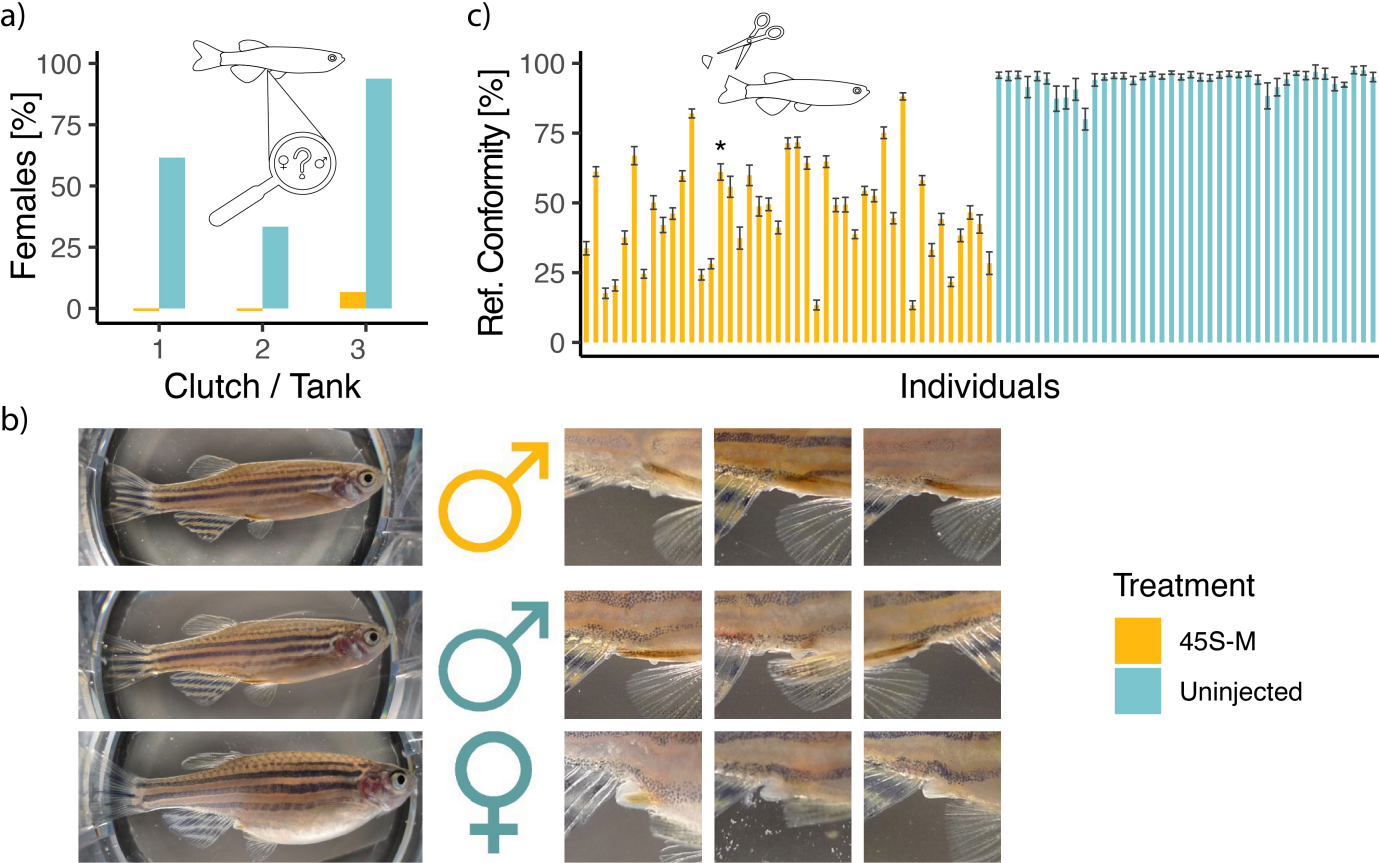
Phenotypic masculinization in response to 45S-M disruption observed at 100 dpf. (*a*) Percentage of females, identified by the presence of a genital papilla, in three clutches from separate parents. (*b*) Typical appearance of treated and untreated fish and examples of the cloacal region with genital papilla clearly visible in females. (*c*) 45S-M promoter reference conformity in fin clips taken off the same individuals. Asterisk marks the single female found in the 45S-M group. Black bars represent margin of error resulting from the number of paired reads.

Besides the strong difference in sex ratios, (male) individuals from the two groups were virtually indistinguishable (figure 3*b*), with no malformations or reduction in growth found among the 45S-M group. A single uninjected individual had developed a cataract in one eye but otherwise appeared healthy. DNA analysis of fin clips, conducted alongside sex assessment, revealed large variability in 45S-M editing in the injected group and a high degree of reference conformity accompanied by low variation in the uninjected group (figure 3*c*).

### (c) 45S-M edited males produce sperm containing modified DNA but are fertile

While the 45S-M edited males appeared phenotypically normal, it was possible rDNA modification might manifest itself in some form of infertility. Crossing several of the modified and unmodified males with different wild-type AB females produced viable F1 embryos that, when sexed at 100 dpf, revealed no further sex skewing (electronic supplementary material, figure S6). This implies either that unmodified 45S-M rDNA from the wild-type mothers can rescue feminization or that modified 45S-M rDNA could not be incorporated into functional sperm and therefore not be inherited.

**Figure 4.**
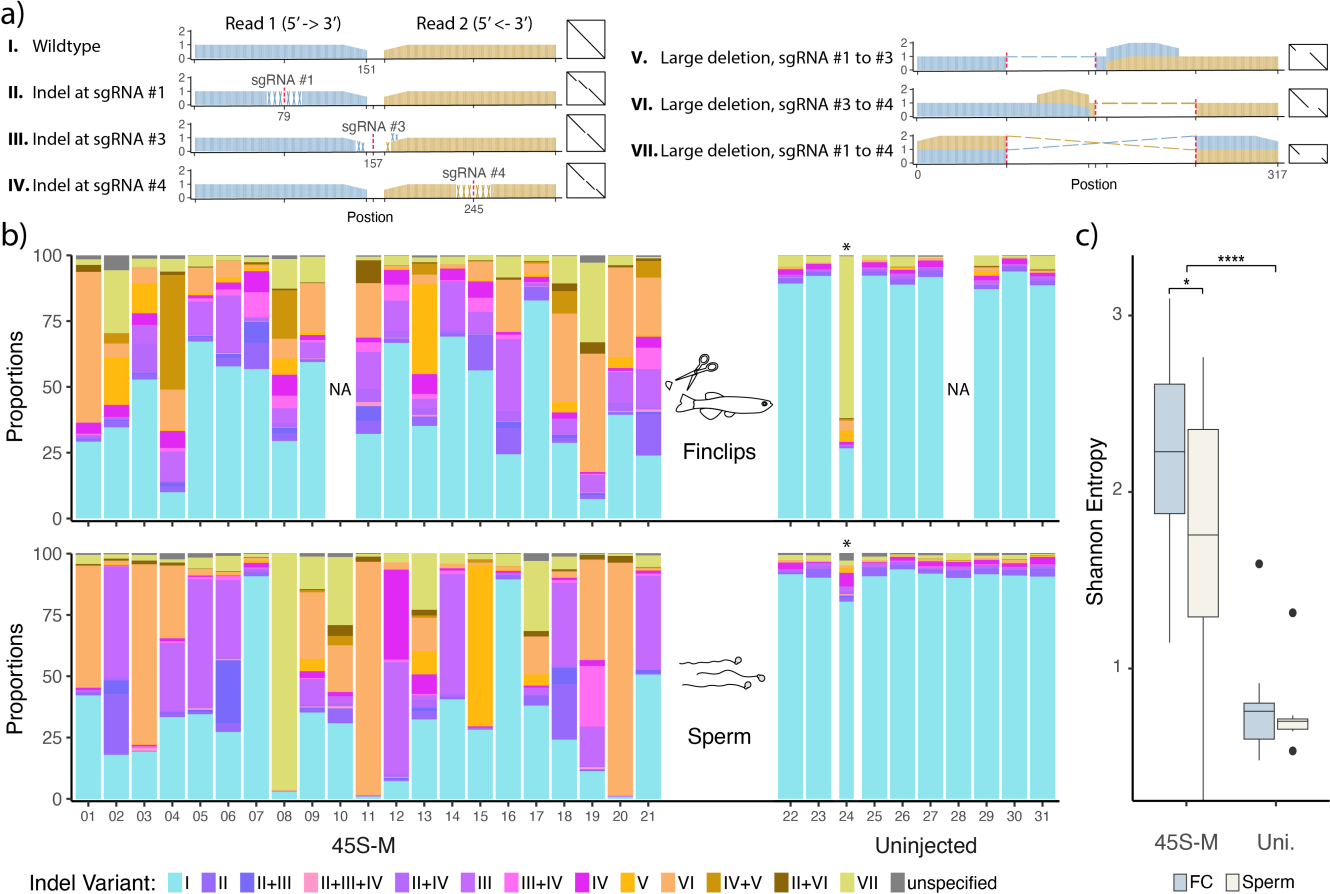
45S-M promoter indel variants. (*a*) The seven indel variant categories caused by the 45S-M knockout treatment with three co-injected Cas9-associated single guide RNAs (sgRNAs), illustrated as relative sequence coverage and dot plots. The proportion of variant I is equivalent to the degree of reference conformity reported in the earlier graphs. PCR amplicons were 151 bp paired-end sequenced, leaving an unresolved 15 bp gap in wild-type reads. sgRNA target loci are indicated in red, cross-hatch patterns indicate indels, and dashed lines indicate large deletions. *(b*) Comparison between indel variant proportions in fin clips taken at 171 dpf and sperm samples collected from the same individuals. Columns not assessed (NA) were rejected for having <150 paired reads. Asterisk marks a 45S-M modified outlier fish that was found amongst the control group individuals. (*c*) Quantification of the observed reduction in indel variant diversity for 45S-M targeted fish, when comparing multi-tissue fin clips (FC) and single-tissue sperm samples, expressed as Shannon entropy. Fin clips and sperm from uninjected (Uni.) control fish are included for comparison. Boxplot minima and maxima (large black dots) indicate outliers. Whiskers are 1.5*interquartile range above and below the 75th percentile and 25th percentile (upper and lower whisker, respectively), which form the bounds of the box, with the centre of the box representing the median. A Wilcoxon rank-sum test was used to calculate the significance of differences between groups (*p ≤ 0.05 and ****p ≤ 0.0001).

To test this, we collected semen and fin clips from all the adult 45S-M edited individuals (*n* = 45). Firstly, microscopical observation of semen revealed freely moving, healthy-looking sperm cells in all samples. We further performed DNA analysis on sperm and fin clips from 21 of 45 samples, finding that both sperm and fin clips were significantly modified, but in a noticeably different manner (figure 4). Because we were using 3× sgRNAs in parallel for targeting, we were able to detect six different classes of mutations (figure 3a), each with unique minor indels at the target site, or larger deletions between them. As before, we found a much higher proportion of affected reads in treated individuals than in the untreated control (figure 3b), with the exception of uninjected sire 24, which had a considerable number of reads not matching the consensus sequence, particularly in the fin clip sample. Interestingly, while having a dramatic effect on oogenesis and feminization of the zebrafish gonad, the large degree (>49%) of 45S-M promoter disruption found in 90% of the semen-derived samples from treated individuals had no effect on spermatogenesis and male fertility. Injected sires 04, 05, 11, 13, 15 and 17 all produced healthy, albeit no longer sex-skewed offspring when bred with wild-type females (electronic supplementary material, figure S6).

While both sample types contained a range of indel variant categories, sperm showed much lower mutation diversity. Indeed, several individuals contained only one or two variant types in sperm; however, they possessed a much greater diversity of mutation classes in fin clip. When this was quantified as Shannon entropy, both the difference between treated and control and also the reduction in diversity from fin clip to sperm was found significant (figure 3c), a result reflecting developmental origin; fin clips arise from many somatic cell lineages, whereas sperm arises from a small number of primordial germ cells early in development.

## 4. Discussion

Here we targeted the promoter of the 45S-M rDNA region with three Cas9-associated sgRNAs in zebrafish zygotes and achieved high editing efficiency across most tested individuals. Individuals affected by the treatment displayed dramatically reduced oocyte-specific 45S-M rDNA compared with presumptive females in the untreated or mock-treated groups and developed almost exclusively into fertile phenotypic males.

### (a) Zygotic mutation of 45S-M does not affect early growth or morphology but drives masculine sexual development

Despite being the overwhelmingly dominant rRNA in the embryo until 1.5−2 dpf [19,44], disruption of the 45S-M promoter did not have dramatic negative effects on early growth or morphology (electronic supplementary material, figures S3 and S4). This lack of early phenotype is likely explained by 45S-M rRNA being maternally supplied, effectively masking 45S-M mutation. Moreover, transcription in zebrafish embryos is silenced across the genome prior to the mid-blastula transition [45], and in unmodified fish, canonical 45S-S rRNA appears to be the only rRNA expressed [19], further isolating embryos from deleterious early effects of 45S-M mutation.

In contrast to the lack of phenotype during early development, we found zygotic disruption of the 45S-M promoter dramatically affected juvenile sex differentiation in independent experiments. All 45S-M edited fish developed into males (*n* = 45) except for one individual, a result entirely unexpected by chance given normal sex ratios in uninjected fish.

Because 45S-M expression is restricted to oocytes [19], it seems likely that it is the germline, and not the soma, that drives the masculinizing phenotype following zygotic editing. Indeed, in zebrafish, it is the germline that holds dominance over hormone-producing somatic cells of the gonad with respect to sex determination [46,47]. Specifically, insufficient ‘type-1B’ oocytes result in transformation of the bipotential ‘juvenile ovary’ into a testis and male differentiation [48]. Type-1B oocytes are defined cytogenetically by an abundance of nucleoli—the structural manifestation of ribosome proliferation [49,50]. We have previously detected amplification of 45S-M rDNA in germline cells of presumptive females [20] and here show it can be measured in whole fish, allowing high-throughput genetic screening of this locus to be used as a proxy for sex determination at earlier stages of development. Considering that 45S-M also exactly overlaps the genomic region most strongly associated with sex in wild zebrafish strains, 45S-M has been proposed to be the primary trigger for sex determination in zebrafish [20,47,51,52]. Our observation that zygotic disruption of the 45S-M promoter suppresses feminization (and in turn 45S-M amplification) lends strong support to this hypothesis and may have implications for other species with apparently cryptic sex determination systems featuring rDNA loci on sex chromosomes.

### (b) 45S-M ribosomal DNA mutation spectrum and the minimal ribosomal DNA complement

Despite being injected into the oocyte, Cas9-derived mutations are well known for being heterogeneous in zebrafish [53], with the endonuclease not always completely effective in the first cell and its activity maintained for at least a few cell cycles post-fertilization. Considering this, it is not unexpected that mosaics produced in our experiments have a spectrum of mutations in some cell lineages, with others unaffected. In line with their smaller progenitor population, sperm show reduced mutational heterogeneity compared with tailfins (figure 3c).

We speculate that the female outlier in the treated group, which showed treatment effects in its fin clip DNA, did not experience modification in the germline. Interestingly, sires 7 and 16 (figure 3b) show this exact mutation distribution across tissues (i.e. mutations presenting in fin clip but not sperm); however, in the same way as many unmodified fish, these sires presumably underwent natural masculinization, despite intact gonadal 45S-M. On the other hand, a threshold level of 45S-M rDNA modification appears to be somewhat tolerated during feminization—heterozygous F1 individuals produced by outcrossing to wild-type fish resulted in no clear sex skewing (electronic supplementary material, figure S6). A future analysis of the female germline in these outcrosses, perhaps using long-read sequencing capable of assessing rDNA sequence across stretches of repeats, may identify the minimal ribosome core required in order to develop and maintain femininity in domestic zebrafish strains. By contrast, long-read sequencing may reveal sequence variations or even differences in repeat copy numbers that are the genetic determinants of the ZZ/ZW sex determination system in wild strains of zebrafish.

### (c) Ribosome heterogeneity and ribosomal DNA specialization

In addition to their implications for sex determination in a flagship vertebrate model, our results provide several points of significance for the (re-)emerging field of ribosome heterogeneity. There are increasing reports of rRNA specialization occurring across a variety of species [6,54]; however, these studies collectively lack proof of functional differences between rRNA variant loci.

Despite this, ribosomes of the zebrafish oocyte and early embryo display several features strongly indicating functional specialization. For example, maternal ribosomes display long periods of translational dormancy, recently shown to be controlled by specific accessory protein factors essential for the creation of high-quality embryos [23,55]. Moreover, these ribosomes need to translate maternally derived mRNAs exclusively, and they need to do so throughout extremely short cell cycles post-fertilization. As such, it seems likely that the significant sequence divergence between zebrafish 45S-M rRNA and its somatic counterpart represents genuine rRNA specialization and cannot be explained solely by variation in rRNA number.

Here we show that not only can the 45S-M rRNA locus be edited on account of its sequence divergence from somatic rRNA but also that 45S-M disruption in zygotes leads to a non-deleterious phenotype (i.e. masculinization) that does not affect growth or subsequent fertility when outcrossed. As such, we contend that 45S-M is the most tractable vertebrate system discovered to date in which to study rDNA specialization. Future experiments focusing on selectively mutating regions of 45S-M or replacing it with the somatic alternative will offer further insights into functional specialization of variant locus and the ribosomes it contributes to.

## Data Availability

Raw sequencing data and custom 45S-M indel variant identification scripts are available under [38]. Supplementary material is available online [56].
